# A mixed methods investigation of end-of-life surrogate decisions among older adults

**DOI:** 10.1186/s12904-020-00553-w

**Published:** 2020-04-02

**Authors:** Eleonore Batteux, Eamonn Ferguson, Richard J. Tunney

**Affiliations:** 1 Department of Clinical, Health and Educational Psychology, University College London, 1-19 Torrington Place, London, WC1E 7HB UK; 2 University of Nottingham, Nottingham, England; 3 Aston University, Birmingham, England

**Keywords:** Surrogate decision-making, Self-other differences, End-of-life, Substituted judgment standard, Mixed methods

## Abstract

**Background:**

A large number of end-of-life decisions are made by a next-of-kin for a patient who has lost their decision-making capacity. This has given rise to investigations into how surrogates make these decisions. The experimental perspective has focused on examining how the decisions we make for others differ from our own, whereas the qualitative perspective has explored surrogate insights into making these decisions.

**Methods:**

We conducted a mixed methods study to bring these two perspectives together. This is crucial to comparing decision outcomes to the decision process. We asked older adult partners to make end-of-life decisions for each other. They then took part in a semi-structured interview about their decision process. Transcripts were analysed using thematic analysis.

**Results:**

24 participants took part in the study. Surrogates were more likely to take a life-saving treatment at the risk of a diminished quality of life for their partner than for themselves. This was consistent with their transcripts which showed that they wanted to give their partner a better chance of living. Although there was evidence of surrogate inaccuracy in the decision task, participants overwhelmingly reported their intention to make a decision which aligns with the substituted judgment standard. However, uncertainty about their wishes pushed them to consider other factors.

**Conclusions:**

Taking a mixed methods approach allowed us to make novel comparisons between decision outcome and process. We found that the intentions of surrogates broadly align with the expectations of the substituted judgment standard and that previous discussions with their partner helps them to make a decision.

## Background

In the event that a patient has lost their decision-making capacity due to illness or injury, it is common for a next-of-kin to take on the role of a surrogate to make medical decisions on their behalf. In the United States (US), at least 70% of intensive care unit deaths are the result of decisions to withhold or withdraw life-sustaining treatment, but only about 5% of patients are able to make these decisions for themselves [1]. If the patient has not written an advance directive, it is usually the case that the next-of-kin will act as a surrogate. Given the increase in age-related illnesses in westernised countries, the need for surrogates is growing [2].

An ethical framework of surrogate decision-making was developed almost three decades ago, of which the underpinning principles remain representative of current legislation in many western countries^1^ [3]. The ethical framework stated that if an advance directive is available, it should be followed. Otherwise, the substituted judgment standard should be applied, whereby the surrogate must decide based on their knowledge of the patient’s preferences – i.e. make the decision that the patient would have wanted. When little is known about the patient’s preferences, the best interest standard applies whereby the option which provides the best possible outcome is chosen. The exact use and implementation of these principles varies. For example, legislation in the United Kingdom (UK) combines the substituted judgment standard with the best interest standard and states in the US are moving towards the best interest standard. Nevertheless, the substituted judgment standard continues to be used and is worth assessing.

The substituted judgment standard assumes that surrogates can accurately predict patients’ preferences and that they are willing to decide based on these predictions. However, concerns have been raised regarding its validity. Firstly, surrogates report distressing experiences due to the difficulty of making a decision they are comfortable with whilst respecting the patient’s wishes [4]. Patients report they would like family members and physicians to input in the decision process, rather than it being solely based on their own preferences [5]. Secondly, the assumption that surrogates can accurately predict patients’ wishes has been heavily questioned. A systematic review found that surrogates can predict their next-of-kin’s treatment preferences around 68% of the time [6]. Moreover, surrogates are biased towards predicting that patients would want to be treated, making them more accurate in cases where patients are favourable to treatment [7]. Finally, even if surrogates had full knowledge of the patient’s preferences, do they decide according to them? In this paper, we aim to further our understanding of how surrogates make these decisions.

### How do surrogates make decisions?

There have been qualitative investigations of surrogates’ experience of making these decisions after they have taken place [4, 8–11]. These show that surrogates do consider the patient’s wishes, either by recalling previous conversations or their shared experiences, but they are not the sole focus of their accounts. Other factors can conflict with deciding in accordance with the patient’s wishes, such as their own values or preserving the patient’s life or the family’s well-being. Indeed, surrogate decisions are often biased towards the decision-maker’s own preferences [12, 13]. Consequently, the substituted judgment standard cannot always be met.

Parallel to this literature, a strand of experimental research has found that we are more likely to choose the option with the lowest risk of death for others [14, 15]. However, for ourselves, we are more likely to choose the option which might increase our chances of dying in order to avoid an illness [16] or complications from taking a treatment [17]. This has been explained by professional accountability when medical professionals make decisions [18]. When the general population decide for a stranger or family member, the reasons are not clear [19–21]. Interestingly, discrepancies have been found between surrogate choices and predictions. Surrogates avoid a risk of death for someone else more than themselves, despite predicting that they have similar preferences [22–24]. This suggests that surrogates override the recipient’s preferences to make a more cautious decision on their behalf which preserves their chances of living.

### Psychological theories of surrogate decision-making

Tunney and Ziegler’s model [2] puts forward that surrogates engage in various forms of perspective taking when making a decision. The importance they give to each perspective depends on the nature of the decision. For end-of-life decisions – highly significant decisions for which the surrogate can be held accountable – the model predicts that surrogates focus on what is required of them. If the best interests standard should be followed, the surrogate will engage in benevolent perspective-taking (i.e. decide based on what the recipient should do). If the substituted judgment standard should be followed, the surrogate will engage in simulated perspective-taking (i.e. predict what the recipient would do and decide accordingly), particularly if they are close to the recipient and know their wishes. However, the surrogate might also engage in an egocentric perspective (i.e. decide based on what the surrogate wants) to preserve their own interest. If the surrogate has incomplete knowledge of the recipient’s preferences, they might rely on a projected perspective (i.e. decide based on what they would do if they were the recipient). Although the model expects a next-of-kin to focus on a simulated perspective, other perspectives might come into play which prevent strict adherence to the substituted judgment standard.

Other theories make predictions regarding when self-other differences occur. The presence of a *hot-cold empathy gap* between the surrogate and the recipient would lead surrogates to underestimate the intensity of the recipient’s emotional state, such as a patient’s pain [25]. The *risk-as-feelings hypothesis* expects reduced emotional reactions to the prospect of a risk when deciding for someone else [26]. Both assume that surrogate decision-making is no different from one’s own decision process – self-other differences are only a reflection of the psychological distance between the surrogate and the recipient, i.e. how close they are to each other [27]. Finally, *social values theory* suggests that social values are the key factor taken into account when making surrogate decisions and self-other differences should arise when taking a risk is socially valued or frowned upon [28]. None of these accounts are able to capture the intricacies of making surrogate end-of-life decisions, which is likely to be a much more complex and reflective process. Tunney and Ziegler’s model [2], in assuming that surrogates engage in perspective-taking, is more able to support an understanding of how surrogates navigate such complex medical decisions. It can conceptualise the tensions felt by surrogates when needing to abide by the substituted judgment standard and is therefore well suited to analysing surrogate decisions.

### The present research

So far, quantitative studies have looked at self-other differences in treatment scenarios, whereas qualitative studies have focused on the experiences of surrogates without taking into account the specific clinical content of decisions made. We do not yet know much about end-of-life scenarios other than qualitative reports taken place after the fact. There is therefore scope for research that can bridge the gap between the decisions that surrogates make and the reasons they give for doing so. In the present study, we investigate how older adults make end-of-life decisions for their partners via a decision-making task and a semi-structured interview. By taking a mixed methods approach, we position ourselves within a pragmatist epistemological framework whereby we accept that qualitative and quantitative methodologies can hold conflicting ontological and epistemological assumptions, but put these aside in our analysis to focus on addressing the research question and its real world implications [29].

We used an expansion design with mixed methods to extend the scope of inquiry to different inquiry components [30], namely the outcome of the decision (quantitative method) and the process of the decision (qualitative method). Our quantitative research question was: are people more willing to accept a life-saving risky treatment for themselves than for their long-term partner? Given previous findings, we expected participants to accept more treatment for their partner, even if that means risking their quality of life. Our qualitative research question was: which perspectives do surrogates take when making end-of-life decisions for their partners? We conducted in-depth semi-structured interviews which we analysed using a thematic analysis. We recruited older adult partners as they are more likely to make these sorts of decisions for each other in the near future. We could then compare participants’ own decisions to the ones their partner made for them to assess whether any inaccuracy is related to failing to take a simulated perspective (i.e. decide according to their partner’s wishes). We integrate results from both methods in our discussion.

## Method

### Participants

We recruited 12 older adult partners (60–80) in long-term relationships from Nottinghamshire, UK. Recruitment methods included the School of Psychology’s community sample, local University of the Third Age (U3A) branches and word of mouth. Participants were either contacted via email or directed to the investigators via email. Participants took part between April and July 2018. All were able to understand and complete the decision-making task and take part in the interview.

### Procedure

Partners were asked to come to the University of Nottingham together. After giving informed consent, they took part in the study in turn, whilst their partner waited in a separate room. Every participant completed the decision-making task first, which was followed by a semi-structured interview. The ethical and medical context in which surrogate decisions are made were not described to participants to avoid biasing their answers. For example, we did not mention the substituted judgment standard at any point. Participants were debriefed together once both had completed the study.

### Decision-making task

Participants completed three scenarios adapted from the willingness to accept life-sustaining treatment (WALT) instrument [31] (see Supplementary File 1). Each scenario depicted a life-threatening situation in which participants are taken to hospital and offered a high-burden treatment course to recover. The probability of the treatment working varied from 90 to 10% in decrements of 10. In each case, participants had to indicate whether they would want the treatment or not. They were told that without the treatment they would not survive. The outcome of the treatment varied: either the treatment works and their current health is restored, or it does not and they die from the illness (death scenario), end up bedbound (functional impairment scenario) or unaware (cognitive impairment scenario). The functional and cognitive impairment scenarios allow us to examine the risk of impaired quality of life participants are prepared to take to for a chance of living. Every participant completed the scenarios once from their perspective (i.e. making decisions for themselves) and once making decisions on behalf of their partner. The order in which participants completed these was counterbalanced.

### Quantitative analysis

To investigate self-other differences, we computed the average between the lowest chance of recovery participants accepted and the highest chance of recovery participants refused: the point at which they were indifferent between accepting and refusing treatment. To assess whether surrogate decisions were accurate, we computed the difference between surrogate decisions and the recipient’s decisions by subtracting the latter from the former and removing the sign. This gave us a value representing how far surrogate decisions deviated from the recipient’s decisions. We consider a result to be statistically significant at *p* < .05. However, as our sample is small, we will also examine effect sizes which can be more meaningful than *p*-values.

### Semi-structured interviews

Participants took part in in-depth semi-structured interviews conducted by the first author (see Supplementary File 2 for interview guide). The questions were open-ended and designed for participants to freely speak about their experience and thought processes in the decision-making task. The questions were centred around three topics: recall of surrogates’ thoughts when making decisions for their partner, further exploration and discussion of their reasoning and experience, and how their surrogates decisions compared to their own decisions. Interviews ranged from 15 to 45 min, were audiotaped and transcribed verbatim.

### Qualitative analysis

We analysed the interview data using a thematic analysis which allows us to identify and analyse patterns in rich detail.. We were guided by an essentialist/realist epistemological approach which reports experiences, meanings and the reality of participants [32]. We interpreted participants’ motivations and experiences in a straightforward manner, assuming a largely unidirectional relationship between their language and the experiences they report. We took a semantic approach whereby themes were identified within the explicit content and meanings of the data, moving from a description to an interpretation of it. Given our interest in understanding the perspectives participants took when making surrogate decisions, we followed a theoretical thematic analysis driven by the forms of perspective-taking laid out in Tunney and Ziegler’s model [2]. This meant that, although we did not start out with an a priori coding frame, our analysis was driven by our theoretical interest and provided a more detailed account of a particular aspect of the data, rather than a rich description of the entire data set. We directed our analysis towards the decisions participants made for their partner, rather than the ones they made for themselves, as our research question was focused on the surrogate decision process.

We followed the analytical steps as laid out in Braun and Clarke [32], After transcribing the interviews, the first author (EB), who is trained in thematic analysis, worked through the data set to generate codes using NVivo. EB then went through the dataset again to check that all extracts that were pertinent to our codes had been identified, collating codes if necessary. Once the list of codes had been generated, the coding was checked with a researcher independent to the study (WD) who is trained and experienced in a range of qualitative analysis methods. WD was given half of the transcripts (*N* = 12). WD coded the transcripts independently, which was then compared to EB’s coding. EB and WD discussed discrepancies and independently revised their coding, after which the kappa agreement score was 0.98. After this process of triangulation, EB sorted codes into potential themes by considering how different codes might combine to form an overarching theme. EB read through the data extracts of each candidate theme to ensure that they formed a coherent pattern. Next, EB considered whether the thematic map formed a coherent representation of the entire data set by reading through it again. Finally, EB named and defined each theme in order to form an accompanying narrative.

## Results

### Participant characteristics

We recruited 12 partners (*n* = 24) who were all in heterosexual relationships. Participant characteristics can be found in Table 1. The proportion of participants with children and grandchildren was inferred and collated from comments participants made during their interviews. It could not be exactly defined as 92% (*n* = 22) mentioned children and 67% (*n* = 16) mentioned grandchildren.

**Table 1:** Participant characteristics

Characteristic	Participants
Age (in years)	Mean = 67.67 (59–81)*
Gender	50% female (*n* = 12)
Relationship to partner	
Length (in years)	Mean = 41 (10–51)*
Marital status	92% married (*n* = 22)
Children	
Children with partner	75–83% (*n* = 18–20)
Grandchildren from partner	42–75% (*n* = 10–18)
Other children/grandchildren	4% (*n* = 1)

*Values in parentheses refer to the range

### Quantitative findings

Given our small sample size, we took measures to ensure that we could draw meaningful conclusions from our findings. We calculated the effect size we could detect from our sample. With a sample of 18 participants, a large effect size (d > 0.80) can be detected with adequate power (>.80) and an acceptable alpha level (<.05) according to G*Power 3.1 [33]. We calculated post hoc power for our statistically significant results. Further, a member of one of the dyads had very specific health preferences, which stood in contrast to the rest of our sample. This was reflected in their own choices and the ones their partner made for them, and spoken about at length during both of their interviews. Given that we had a small sample, we decided to exclude this dyad from our quantitative analyses to preserve statistical validity. This did not have an impact on the direction of the results, as can be seen in our analyses with the full sample in Supplementary File 3.

#### Self-other differences

**Fig. 1:**
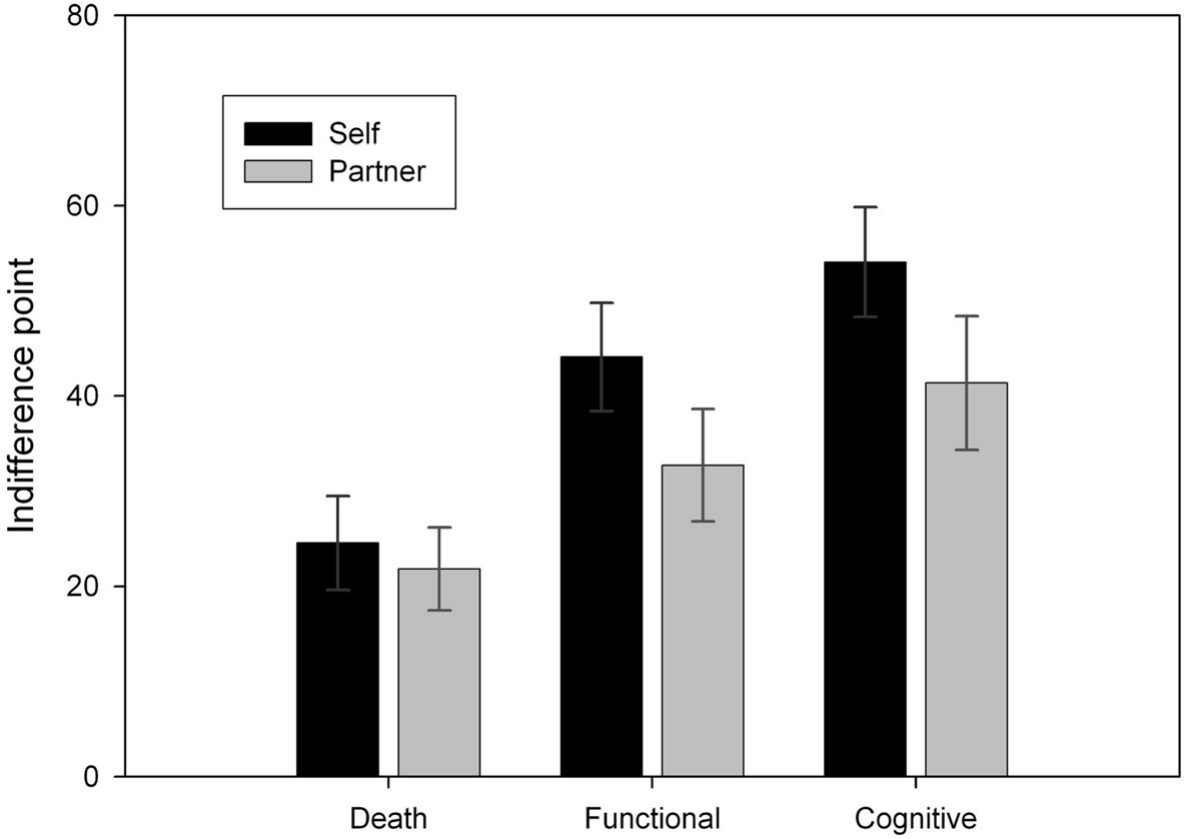
Participants’ indifference points for themselves versus their partner for each treatment outcome. Lower values indicate that participants were willing to accept a treatment with a lower chance of recovery and a higher risk of reduced quality of life. Error bars represent the standard error of the mean

Participants’ indifference points were entered into a 2 (recipient) × 3 (outcome) repeated-measures ANOVA (see Fig. 1). The main effect of recipient was significant (*F*_*1,21*_ = 9.751, *MS*_*e*_ *=* 270.455, *p* = .005, *η*_*p*_^*2*^ *=* 0.317)^2^: a simple effects analysis showed that participants accepted treatment more often for their partner than for themselves (mean difference = − 8.939, *p* = .005). The main effect of outcome was also significant (*F*_*2,42*_ = 22.537, *MS*_*e*_ *=* 299.747, *p* < .001, *η*_*p*_^*2*^ *=* 0.518).^3^ According to a simple effects analysis, participants were overall more likely to accept treatment in the death scenario than in the functional impairment scenario (mean difference = − 15.227, *p* < .001) and the cognitive impairment scenario (mean difference = − 24.545, *p* < .001). They were also more likely to accept treatment in the functional impairment scenario than in the cognitive impairment scenario (mean difference = − 9.318, *p* = .006). The interaction between recipient and outcome approached significance (*F*_*2,42*_ = 2.725, *MS*_*e*_ *=* 118.723, *p* = .077, *η*_*p*_^*2*^ *=* 0.115). Surrogates seem to be more willing to accept a treatment for their partner than for themselves, even if it can reduce their quality of life.

#### Surrogate accuracy

**Fig. 2:**
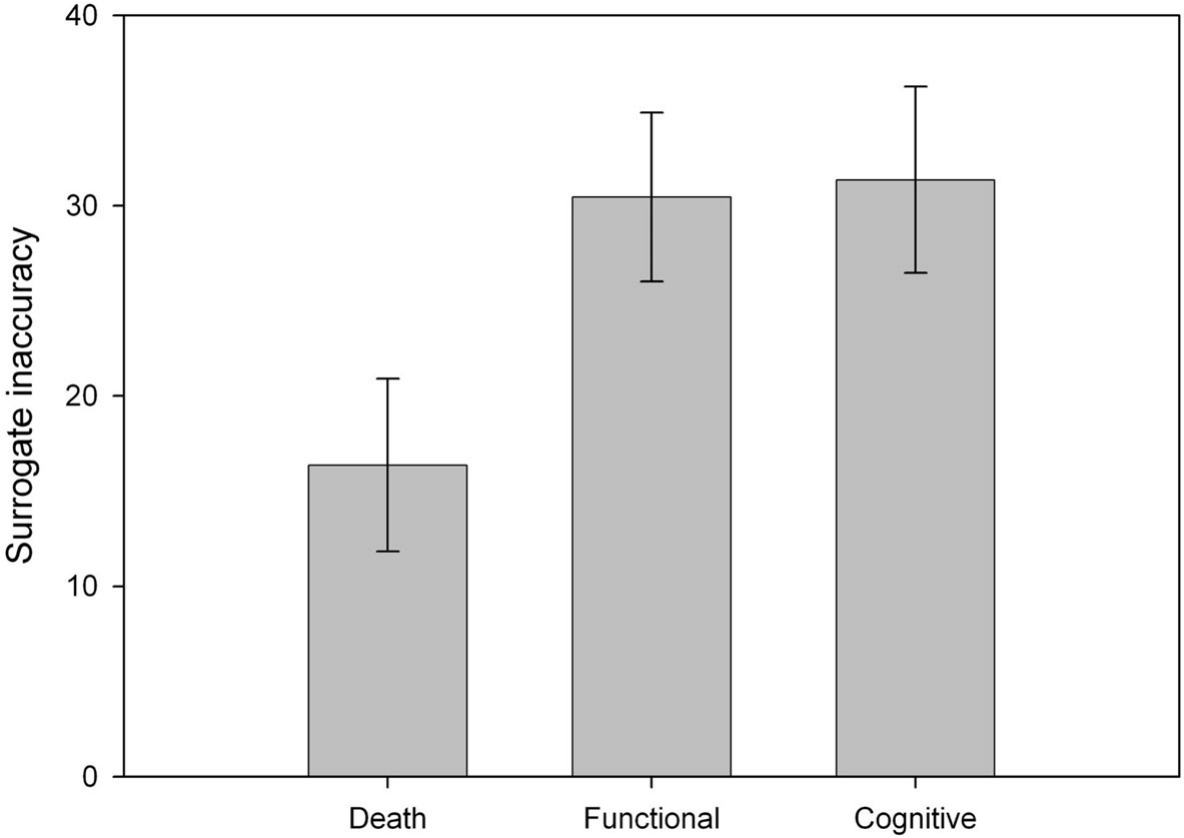
Surrogate inaccuracy represents the deviation in indifference points between surrogate decisions and the recipient’s decisions. Error bars represent the standard error of the mean. Higher values indicate that surrogate decisions were less accurate

We examined whether surrogate accuracy significantly deviated from 0 using one-sample t-tests (see Fig. 2). This was the case for death (*t*_*21*_ = 3.607, *p* = .002), functional impairment (*t*_*21*_ = 6.864, *p* < .001) and cognitive impairment (*t*_*21*_ = 6.410, *p* < .001) scenarios. To investigate whether accuracy differed by scenario, we conducted a repeated-measures ANOVA with outcome as a three-level factor. We found a main effect of outcome (*F*_*2,42*_ = 4.596, *MS*_*e*_ *=* 338.600, *p* = .016, *η*_*p*_^*2*^ *=* 0.180). Pairwise comparisons showed that accuracy in the death scenario was higher than in the functional impairment scenario (mean difference = − 14.091, *p* = .017), as well as higher than in the cognitive impairment scenario (mean difference = − 15.000, *p* = .026). Accuracy between the functional and cognitive impairment scenarios did not differ (*p* = .854). These results indicate that surrogate decisions were less likely to be accurate when the outcome involved living with a reduced quality of life rather than death.

### Qualitative findings

**Fig. 3:**
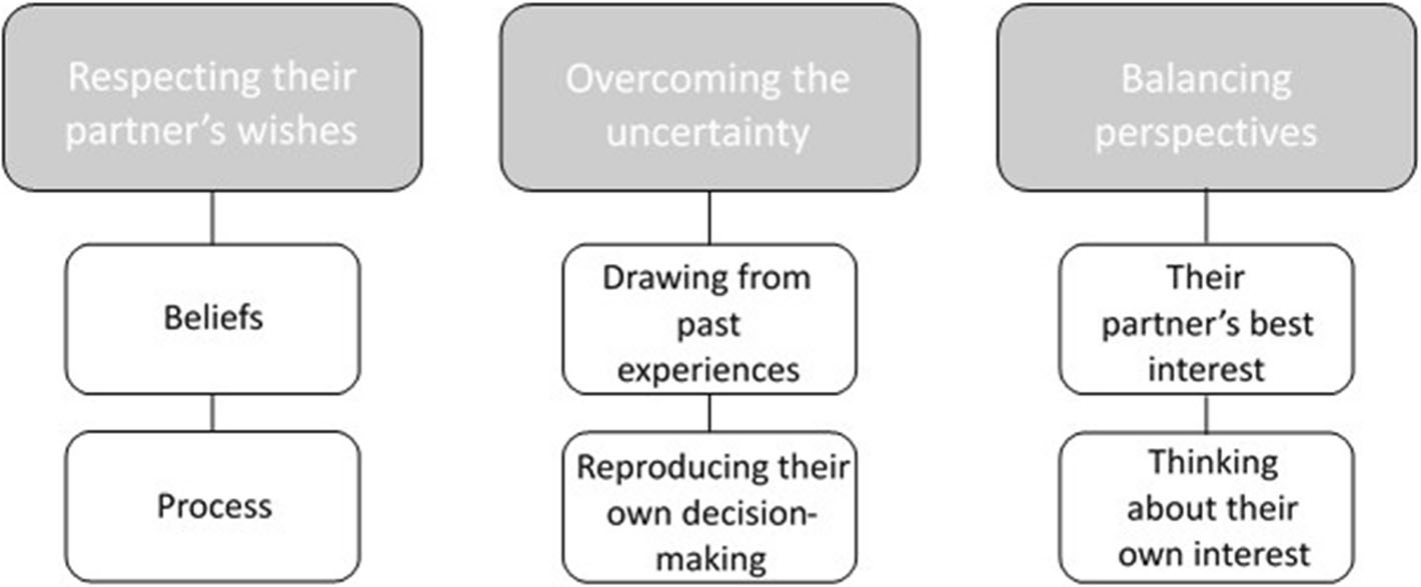
A map of the theme structure generated by our analysis of participants’ interviews

We identified three themes, each composed of two sub-themes: respecting their partner’s wishes (with sub-themes ‘beliefs’ and ‘process’), overcoming the uncertainty (with sub-themes ‘drawing from past experiences’ and ‘reproducing their own decision-making’) and balancing perspectives (with sub-themes ‘their partner’s best interest’ and ‘thinking about their own interest’) (see Fig. 3).

#### Respecting their partner’s wishes

Participants expressed their views that surrogate decisions should be made in line with the patient’s wishes. These translated to their decision-making, as surrogates overwhelmingly identified that their decisions were guided by knowledge of their partner’s wishes. We split this theme into two components: (a) beliefs and (b) process.

#### Beliefs

Participants often made their belief clear that these decisions ought to be made in accordance with the patient’s wishes. It was at times explicitly stated about the decisions they made for their partner, but also expressed in relation to surrogate decision-making in general, suggesting that it is strongly held and internalised: *“You’re sort of their representative in the thinking world really, and you do what you think they would want.”* Participants often believed the right decision was a decision their partner would want: *“The wrong decision would be if you’ve taken an opportunity for them to live in a way they wouldn’t want to live, and you’ve given it to them.”*

Participants highlighted that partners should discuss these scenarios so that they are prepared if they happen and able to make a decision that respects their wishes: *“Well yes I do [think would be the best person to make it], because it’s something that we’ve discussed. […] I do think that people need to talk about these things, and I think there’s this big taboo about people talking about this.”* Almost all participants stated that they should be the designated surrogate because they know the recipient best.

Thinking that surrogates should know the recipient’s wishes highlights the implicit belief that surrogate decisions should be made according to these wishes. A few participants stated that medical professionals should not make these decisions because they do not know the patient well enough: *“If you talk to one another, you know. They know that you’ve always been active and run up the hills, and done this and cycled, and swam or whatever. And you’re still doing it. And there’s a 90% chance that you’re going to end up paralysed and lying in a bed. There’s no way you’d want that. But the doctor might say ‘well, you know, I’ve got to save a life’. […] So you’ve got to be able to say to the doctor they wouldn’t want their life saving.”*

#### Process

Most participants’ intention was to make a decision that respected their partner’s wishes. This was spontaneously reported by participants and usually constituted their answer to the first question posed to them (i.e. to walk the interviewer through their thought process). They often explicitly stated that they reflected on what they thought their partner would want in order to make a decision for them: *“I was trying to imagine if it wasn’t me making the decision, but it was their independent decision, what they would like to happen. And obviously I would make that decision for them based on those thoughts.”* Participants usually referred to their partner’s wishes to justify their decisions. Many spoke at length about what their partner would want in each scenario and based their decisions on this.

Even when participants did not know their partner’s wishes on the matter, they sometimes tried to make a judgment based on their wider knowledge of their partner: *“My partner is the type of person who would definitely want to try everything. […] So I just ticked everything, it’s as simple as that. I don’t know his thoughts on that at all, because he’s a man who never talks about anything personal, so I have no idea on his thoughts and ideas of anything like that at all, so I’ve just had to guess.”* This suggests a three-step process to their decision-making: having the intention to decide according to what their partner wants, recalling knowledge of their partner’s wishes, and searching for other clues in their partner’s thoughts and behaviours that might be indicative of their wishes. For partners who might not discuss their health preferences, they might not be able to recall knowledge of their partner’s wishes and have to rely on step 3 to infer their wishes.

In terms of how participants felt towards this process, one mentioned they would not feel guilty because they knew they chose what their partner would want, whereas another felt guilty for not doing so. Participants also felt more confident about their decisions when they knew they had taken their partner’s wishes into account. This made their decision process easier and made them feel like they made the right decision: *“I’m quite confident that he’d be the same, that he would feel that I’m making the right decisions. […] Because that’s how we believe life is, you know. We don’t want to survive if we’re mentally or physically, you know.”* Conversely, when comparing the process of deciding for their partner to the process for themselves, participants often mentioned that it was more difficult for their partner because they were less sure of their partner’s wishes than their own: *“What if you got it totally wrong? What if they wouldn’t mind being stuck in that bed and they’d take a 10% chance of it working alright? And 90% chance of being stuck in a bed? What if they wouldn’t mind that but you said ‘let them die’. […] I think I’d know for myself at what point I would cut off, I’d say ‘no this is enough’. Weighting it all up, the effect on my family, the effect on me, I’d know. But I can’t see inside somebody else’s head. I can’t can I. I can assume I know what they want, even though I’ve known her a few years. I can’t guarantee it.”*

#### Overcoming the uncertainty

Participants overwhelmingly expressed the burden that surrogate decisions involve. Despite feeling like they knew their partner’s wishes, that there was still a level of uncertainty. One spoke of there always being a ‘*nagging doubt*’, whereas their own decisions were ‘*cast-iron*’. Participants stressed it was difficult to judge the level of risk their partner would be prepared to take, making the cut-off point challenging to establish: “*The one that was quite challenging was where do you cut off the risk of the treatment not working and leaving somebody stuck in bed but aware? What sort of cut off level? When you’ve got 20% chance of walking out here fit and well, if we don’t treat you you’re going to die, if we do treat it there’s an 80% chance of you ending up in that bed there, and having it out again. And you’re thinking, you know, 1 in 5, is it worth it? 1 in 10, is that worth it? Is it worth trying? That was difficult.”* Participants had to resort to other sources of information to guide their decision-making and ease the process: (a) their past experiences and (b) their own decision-making.

#### Drawing from past experiences

Participants drew from a pool of past experiences they shared with their partner to inform their decision-making, namely life events and discussions concerning illness and end-of-life care. Many spoke of their close relatives or friends who had experienced reduced quality of life and expressed theirs and their partner’s strong wish to not find themselves in those states. This seemed to shift their focus towards quality of life when making decisions, rather than the mere preference for life itself: *“I think if she was very ill, and ended up having her quality of life reduced as such that she couldn’t get out the house, or walk, or ride a bike, or do anything, she’d want to die. She’d say ‘I’ve got no life, I’ve got no quality, I’ve got nothing’. […] Looking after her father that’s 80 something. She’s having to go out and sort him out, all the time. […] And she’s seen it from that end, and she’s seen what it does to other people. So I don’t think she’d do it.”*

On occasion, participants had been surrogates for family members, which they reported influenced their decisions. A few had worked in the care system and had witnessed patients at the end-of-life as well as families having to make these decisions. These experiences shaped their outlook on end-of-life care, which made them more aware of these issues and likely to discuss them with their partner, which was identified as guiding their decision-making: *“So many people don’t [discuss this]. […] there’s a lot of people out there that don’t even go there. Don’t think about it, the consequences. Until you’re in… But I think because we think ahead, so you think that it’s easier to make that decision.”*

Recalling these discussions reassured them and confirmed that they were making an accurate decision, which eased the decision process: *“We’re in a good position because we’ve talked about it before, so decisions are somewhat easier than if it just came out of the blue and then I’d have to decide.”* They anticipated they would give them the courage to make that decision in real life: *“A lot of the decisions that are made are to prolong a person’s death, and then they’re not given a comfortable death at the end of it, because people are too frightened to take the decisions on their behalf, because they would feel guilty. Whereas it’s something that we have discussed, between ourselves and with our children.”* However, there were cases where participants had not had these discussions and would not feel up to making these decisions: *“It is like ‘do you pull the plug on life support’. No I couldn’t. I couldn’t. To me it would be like shooting him, stabbing him, I can’t do that. I couldn’t do that to anybody. And that’s really hard. Had he told me his thoughts before, then that would be something different.”*

#### Reproducing their own decision-making

Many participants reported sharing similar views to their partner regarding end-of-life, meaning they could refer to their own thoughts to inform their judgment about their partner’s: *“I know that the reason that I put the same for him as I would for me, is because that’s how he would feel in the same way. So we’d both do similar things I think. It’s just where the cut off would be”*. Most mentioned they did not consider each scenario to be equivalent, both for them or their partner. Their own reasoning for each scenario was therefore applied when making surrogate decisions: *“On the first page, the treatment, you know, it will either work or it won’t work. Well yes, you go for everything. If it’s there, go for it, there’s always a chance. The second one where you would be bedbound, you’ve still got a life, and I would always be there to look after him, if not there would be other people… We have family. And I think both of us want to see our grandchildren grow up, whatever, so the bedbound bit, yeah, go for it, really. Because it could work, that’s the thing, it could work.”* Finally, participants’ judgment about the risk of the treatment not working (i.e. not leading to a complete recovery) was applied to both their own and surrogate choices. Interestingly, individual participants reported very different judgments about what an acceptable level of risk is, as depicted in Table 2.

**Table 2:** Judgments about the risk of the treatment not working across participants

High risk	*“10% chance is still a chance, so you’ve got to take that chance. If you say 90% chance you might be bedbound, well fine we can always get assistance to help you with that. The ultimate thing is, without the treatment, you’re gone, so a 10% chance has to be taken really.”*
Medium risk	[speaking about the surrogate decisions] *“I think on all the questions I went down to the 50/50, and that would be my final gamble. If it was 50% chance, you might as well take it. Less than 50, I just said no.”*
Low risk	*“I think, I sort of, more for the physical, I probably gave, slightly… I mean I think it was only 80/90, sorry 80/90% chance you get a full recovery. But otherwise, no. Because I think then you’re getting into the realms that, you know, you’re getting the higher risk chance that you are.”*

#### Balancing perspectives

Most participants were also driven by other factors than their partner’s wishes, even if they did consider them. This meant that they had to find the right balance when making their final decision: *“I think it’s weighing up the risk, and your wishes and your partner’s wishes.”* Having to balance these different perspectives implied that they thought more about their surrogate decisions than their own and incorporated more factors into their decision process: *[asked about any difficulties] “No, not for myself, no. Think that was simple enough. […] There was a lot more thought [for my partner], more thought came into it. I thought would the girls want me to do, my two daughters, things like that you know. Then I thought about the wife again – well she wouldn’t want that anyway. Things like that.”* Participants were particularly keen to consider: (a) the partner’s best interest and (b) their own interest.

#### Their partner’s best interest

Participants mentioned having their partner’s best interest at heart, which led them to consider the option most beneficial to their partner’s quality of life. Occasionally, they were more focused on a benevolent than a simulated perspective. Not letting their partner suffer was particularly important. In fact, participants sometimes considered the right decision as dependent on its outcome, rather than the way in which it was made: *“You might admit the wrong decision if it turned out that she would be in a terrible state, you know bed-ridden, in a dire nursing home where there’s no nurses to clear her up.”*

Overall, participants were more willing to have their partner treated than themselves. Deciding about someone else’s life, rather than their own, meant that they really did not want to get it wrong. It pushed them to give their partner a better chance of living: *“I was more inclined to let them have the treatment. Just to give them a fighting chance. But for myself, no.”* A few mentioned that the mental capacity of their partner would affect their decision. They referred to conflicts between a relative’s past and present wishes, in cases where they had lost their decision-making capacity. In such situations, participants might override their partner’s wishes to make a decision that is in their best interest: *“So I think you have to respect decisions, but there comes a point maybe, if my wife said she wanted to be resuscitated indefinitely on a dementia ward, you’d have to say ‘I think it’s time to override that’. […] She’d have to have gone past the point of being able to make a rational decision.”*

Finally, participants insisted on taking into account the wishes of the family. These decisions obviously have consequences for them and they thought they should be consulted and considered during the decision process: *“I had to really sit and try and imagine, you know, could I go through that, could his family go through the fact it didn’t work. The risk of the disruption to our lives and family’s lives, because we have someone in that predicament.”*

#### Thinking about their own interest

Participants clearly did not want to lose their partner and were impacted by that prospect when making their decisions: *“I think we’ve both got the same concerns of being alive and immobile. So that leaves the same worry. But selfish reasons may push me on to have her treated at worse odds than what I would. But that would be selfish reasons again. Nothing else.”* This feeling was quite strong in a couple of participants who chose to treat their partner a lot more than themselves: *“I would go for a much lower percentage with him, and that’s purely emotional because I don’t want to lose him. I’m willing to risk that lower percentage, but I don’t think he would.”* Some mentioned that the emotional turmoil of the decision in real life would make it even harder for them to reject a life-saving treatment: *“I suspect when you’re faced with it, life is very precious. Fear of death is very real.”*

Participants often had to weigh their own wishes against their partner’s, making it difficult to strike the balance. This led them to make a decision in line with their partner’s wishes, but slightly adjusted to give them a better chance of living: *“I know he would not want to live a very restricted life, or if he didn’t have his mental faculties, he wouldn’t want that. But then if there’s still a chance of, you know, a recovery if you like, I think I would want that. So it’s weighing that up.”* Participants also considered the impact that their partner’s illness would have on their own life. This sometimes tipped the balance the other way in favour of taking less treatment, because a functional or cognitive impairment might be too burdensome on them and the family.

Participants occasionally viewed the responsibility placed on them as a burden, but were prepared to take that responsibility and stand by their decisions: *“These are big decisions and I think you’d certainly be accountable and responsible for it. You might have regrets but at least you could look bad and say ‘well I did this in her best interests’.”* However, they were sometimes inclined to let their partner be treated to a greater extent than themselves because of that responsibility: *“For him, I would say have the treatment, have it have it have it. It could work, it could work, and that would always be… I wouldn’t want to be the one to say ‘no’.”* That responsibility was occasionally mentioned as what made the decision process more difficult for their partner than for themselves: *“Because once you’re gone you’re gone, and I can’t bring that back. And if I’m the one who’s making the decisions on his behalf, then that’s almost even trickier I think, because you’re making the decision for somebody else, and you’ve got to live with that as well.”*

Although most made references to an egocentric perspective, it was not usually prioritised over their partner’s wishes. They were still capable of setting aside their selfish motives in cases where they strongly conflicted with their partner’s wishes: *“If I was purely, if I was very selfish and just thought of me, it would be totally different. […] I have different attitudes to it, the thought of somebody being bedridden and have not compos mentis at all, I think that’s pointless.”* Crucially, it was recognised that knowing what their partner would want makes it easier to avoid falling into deciding based on selfish reasons: *“If I didn’t know what I know, then obviously you’d fight to save your partner’s life wouldn’t you. To keep them with you. But I know that’s not what he wants.”*

## Discussion

### Bringing our findings together

Participants were more inclined to accept a life-saving treatment for others than for themselves, which is in line with previous research showing that people are more likely to favour a life-saving choice for others. This is concurrent with participants reporting they did not want to lose their partner and felt that they should give them a chance of living. We found discrepancies between surrogates’ choices and those made by the recipient, indicating evidence of surrogate inaccuracy. However, these results alone do not indicate whether participants intended to go against their partner’s wishes: some might have made a best-informed guess from their knowledge of their partner but got it wrong, whereas others could have known what their partner wanted but chose to make a different decision. Indeed, surrogates reported taking a variety of perspectives to inform their decision-making. Nevertheless, the majority of surrogates intended to decide according to their partner’s wishes and held beliefs that aligned with the ethical underpinnings of the substituted judgment standard.

Most participants held the view that they would rather die than end up with a severely compromised quality of life, and showed that they knew their partner did too. Crucially, this indicates that the source of surrogate inaccuracy might not reside in the fact that surrogates misjudged their partners’ preferences concerning the choice outcomes, but rather that they misjudged their risk preferences. Surrogates did find the cut off level difficult to judge for their partner. Although the reports showed that participants overall held similar views regarding quality of life, they held quite different intuitions regarding the percentage risk that would be ‘too risky’. It is likely that they believed their partner would hold the same intuitions and would only majorly adjust their risk preference if they thought their partner’s wishes differed from their own.

Using a mixed methods approach has enabled us to consider both the process and the outcome of the decision in greater depth. The investigation of self-other differences on their own is nowhere near sufficient to understand surrogate decision-making. Drawing from participants’ reports is necessary to address the complex processes at play. Similarly, identifying how participants’ reports match up to the decisions they made allowed for further nuance in understanding their decision process.

### Theoretical implications

Our findings lend support to predictions made by Tunney and Ziegler’s model [2] (detailed in the Background section). Indeed, we found that surrogates intended to make a simulated decision for their partner in end-of-life scenarios. However, no matter how well surrogates felt like they knew their partner’s wishes, there was a remaining level of uncertainty for some which they had to overcome. They therefore drew on their own decision-making, which is in line with Tunney and Ziegler’s [2]prediction that surrogates might default to a projected perspective. Moreover, participants considered a benevolent perspective by thinking about whether the treatment they would put their partner through was in their best interest. Finally, participants took an egocentric perspective when thinking about their own wishes for their partner.

It is clear that surrogate decisions can be a lot more complex than suggested by the theories we outlined in the introduction, namely hot-cold empathy gaps [25], the risk-as-feelings hypothesis [26] and social values theory [28]. Even though self-other differences might seem like they can be explained by these accounts, neither are able to capture the complexity of the decision process and the intricacies across surrogates’ experiences. Tunney and Ziegler’s model [2] is far more able to highlight these details.

### Practical implications

Participants spontaneously indicated a willingness to honour their partner’s wishes, meaning that the fundamentals of the substituted judgement standard are not necessarily misguided and should not be done away with as an ethical framework. However, the problems previously raised about the substituted judgment standard were highlighted in our study. Surrogates found it difficult to ignore other factors, such as what they want for their partner. A few mentioned that if their partner’s present wishes were considered unreasonable or discordant with their past wishes, they would override them. Expecting the surrogate to make a decision they deem unreasonable magnifies the burden placed on them. Participants also conjectured that it would be more difficult to follow in actual fact due to emotional influences, which is reflected in studies of surrogate decisions after the fact [4, 8]. The present study therefore shows that, in principle, surrogates would like to follow the substituted judgment standard, but this is not always achievable in practice.

Another problem with the substituted judgment standard is that it is entirely focused on the patient and does not address the burden on the decision-maker. Participants who had not had previous discussions with their partner were in the dark about their partner’s wishes, whereas those who had were more confident and comfortable with their decisions. Encouraging people to have these discussions in the later part of their lives would be a good strategy to ease the process. Indeed, a retired health care professional insisted that these discussions between family members do not happen enough prior to the event. Promoting advance care planning practices could be a way to encourage people to discuss their healthcare preferences with their loved ones. To be most effective, it should consider factors which can help surrogates make decisions in all kinds of scenarios, such as the chance of recovery following treatment as our study shows that partners may not be aware they have different risk preferences.

### Limitations

Our study used hypothetical scenarios. Although some participants were certain they would make the same decisions in real life, it was apparent that others were not. Some mentioned that the fear of death would be more imminent in a real scenario, and although they would like to think that they would stick to their current decisions, they felt that they might be led towards a different direction. Indeed, in reports which took place after the fact, the tensions felt by surrogates which prevented them from honouring the recipient’s wishes were more apparent [4, 8, 9].

Our findings might not be easily generalisable to the wider population. It is conceivable that the surrogate decision differs by demographic (education, socioeconomic status, religion etc). Although we did not collect extensive demographic information, it can be inferred that participants were numerically literate given that they were all able to complete the task which involved making judgments about probabilities. Moreover, most participants were open to speaking about end-of-life. The recruitment process clearly stated that the study would involve thinking about severe illnesses and death, which would have discouraged those unwilling to do so. The participants we recruited would presumably be more likely to have these discussions with their partner. Participants who are less numerically literate and open to speaking about end-of-life might have made different decisions. Future research should be extended to different populations to investigate whether their decision process is different to our findings.

## Conclusions

Taking a mixed methods approach enabled us to bring together two facets of surrogate decision-making and their respective literatures. Surrogates did believe that end-of-life decisions for their partner should respect their partner’s wishes, which suggests that the substituted judgment standard is not necessarily inadequate. On the other hand, it is clear that surrogates also incorporate other perspectives in their decision-making and cannot entirely put their own wishes aside. We showed that manifestations of surrogate inaccuracy are not necessarily due to surrogates failing to decide according to their partner’s wishes. Instead, it could be due to individual differences in risk preferences rather than simply a misjudgement of the recipient’s wishes. Future research should further explore the sources of inaccuracy to help surrogates make better informed judgments about their partner’s wishes. Finally, our study suggests that surrogates draw from prior discussions with their partner, which give them the confidence that they are making the right decision. Future work should investigate whether encouraging families to speak about end-of-life makes the process less conflictual, distressing and uncertain.

## Supplementary information

**Additional file 1: **Supplementary File 1. *WALT instrument.*

**Additional file 2: **Supplementary File 2. *Interview guide.*

**Additional file 3: **Supplementary File 3. *Quantitative analysis with full sample.*

## Data Availability

The quantitative dataset used and analysed during the current study is available from the corresponding author on request. The full qualitative dataset is not available to avoid compromising individual privacy.

## Abbreviations

WALT: Willingness to accept life-saving treatment
U3A: University of the Third Age
UK: United Kingdom
US: United States

## Footnotes

1 The specific legislation regarding the conditions under which surrogate decision-making occurs and the procedure it follows varies from country to country, although similar ethical principles are applied in westernised countries.

2 According to a post hoc power (PHP) analysis, a sample of 22 participants was sufficient to detect an effect size of *η*_*p*_^*2*^ *=* 0.317 with PHP > 0.95.

3 According to a post hoc power (PHP) analysis, a sample of 22 participants was sufficient to detect an effect size of *η*_*p*_^*2*^ *=* 0.518 with PHP > 0.95.
