# Supplementary material for: A mixed methods investigation of end-of-life surrogate decisions among older adults
Source: BMC Palliat Care. 2020 Apr 2;19:44. doi: 10.1186/s12904-020-00553-w (PMC7119279; doi:10.1186/s12904-020-00553-w)
Supplement: Supplementary file 1 — Additional file 1: Supplementary File 1. WALT instrument. [file 12904_2020_553_MOESM1_ESM.docx]

WALT instrument

*Decisions for the self*

*Death.* Think about your current state of health. Now imagine that you are suddenly sick with an illness that requires you to be in the hospital for weeks to months. In the hospital, you are offered treatment. The treatment includes many minor tests, such as x-rays, blood draws and CT scans. You also need major therapies such as being in the intensive care unit, receiving surgery, or having a breathing machine. Without the treatment, you will not survive. The doctor tells you there is a X% chance that the treatment will work and get you back to your current state of health and a X% chance that it will not work and you will not survive. Do you want the treatment?

*Functional impairment.* Think about your current state of health. Now imagine that you are suddenly sick with an illness that requires you to be in the hospital for weeks to months. In the hospital, you are offered treatment. The treatment includes many minor tests, such as x-rays, blood draws and CT scans. You also need major therapies such as being in the intensive care unit, receiving surgery, or having a breathing machine. Now imagine that at the end of the treatment, you would be in a state where you would be bedbound. You would not be able to get up out of bed to the bathroom by yourself, and you would need help with all of your daily activities. Without the treatment, you will not survive. The doctor tells you there is a X% chance that the treatment will work and get you back to your current state of health and a X% chance that it will not work and you will be bedbound. Do you want the treatment?

*Cognitive impairment.* Think about your current state of health. Now imagine that you are suddenly sick with an illness that requires you to be in the hospital for weeks to months. In the hospital, you are offered treatment. The treatment includes many minor tests, such as x-rays, blood draws and CT scans. You also need major therapies such as being in the intensive care unit, receiving surgery, or having a breathing machine. Now imagine that at the end of the treatment, you would be in a state where your mind would not be working, such that you would not be aware of what was going on around you or be able to recognize your loved ones. Without the treatment, you will not survive. The doctor tells you there is a X% chance that the treatment will work and get you back to your current state of health and a X% chance that it will not work and you will be unaware. Do you want the treatment?

*Decisions for partner*

*Death.* Think about your partner’s current state of health. Now imagine that they are suddenly sick with an illness that requires them to be in the hospital for weeks to months. In the hospital, they are offered treatment. The treatment includes many minor tests, such as x-rays, blood draws and CT scans. They also need major therapies such as being in the intensive care unit, receiving surgery, or having a breathing machine. Without the treatment, they will not survive. The doctor tells you there is a X% chance that the treatment will work and get your partner back to their current state of health and a X% chance that it will not work and your partner will not survive. Would you choose the treatment for them?

*Functional impairment.* Think about your partner’s current state of health. Now imagine that they are suddenly sick with an illness that requires them to be in the hospital for weeks to months. In the hospital, they are offered treatment. The treatment includes many minor tests, such as x-rays, blood draws and CT scans. They also need major therapies such as being in the intensive care unit, receiving surgery, or having a breathing machine. Now imagine that at the end of the treatment, they would be in a state where they would be bedbound. They would not be able to get up out of bed to the bathroom by themselves, and they would need help with all of their daily activities. Without the treatment, they will not survive. The doctor tells you there is a X% chance that the treatment will work and get your partner back to their current state of health and a X% chance that it will not work and your partner will be bedbound. Would you choose the treatment for them?

*Cognitive impairment.* Think about your partner’s current state of health. Now imagine that they are suddenly sick with an illness that requires them to be in the hospital for weeks to months. In the hospital, they are offered treatment. The treatment includes many minor tests, such as x-rays, blood draws and CT scans. They also need major therapies such as being in the intensive care unit, receiving surgery, or having a breathing machine. Now imagine that at the end of the treatment, they would be in a state where their mind would not be working, such that they would not be aware of what was going on around them or be able to recognize their loved ones. Without the treatment, they will not survive. The doctor tells you there is a X% chance that the treatment will work and get your partner back to their current state of health and a X% chance that it will not work and your partner will be unaware. Would you choose the treatment for them?
