# Supplementary material for: A mixed methods investigation of end-of-life surrogate decisions among older adults
Source: BMC Palliat Care. 2020 Apr 2;19:44. doi: 10.1186/s12904-020-00553-w (PMC7119279; doi:10.1186/s12904-020-00553-w)
Supplement: Supplementary file 2 — Additional file 2: Supplementary File 2. Interview guide. [file 12904_2020_553_MOESM2_ESM.docx]

Interview Guide

*I. Thought processes and experiences when making surrogate decisions*

- I would like you to think back to when you were making decisions for your partner. Could you walk me through that process?
- What thoughts did you have? Did you picture the possible consequences in your head? Were there any points where you were hesitating?
- What was the most important factor for you? Any particular information in the scenarios that drove your decision? Did you consider anything else?
- What was the most challenging?

*II. Discussion of surrogate decisions*

- How do you feel about the decisions that you made? Do you think you made the right decisions? What makes you think you made the right decisions (or not)?
- How do you see your role in making these decisions for your partner? Would you feel responsible for the outcome? Do you think that affected your decisions?
- Do you think these decisions are what your partner would want? What would be the most important factor for your partner?
- Would you want to be the person making these decisions for your partner? Would you be the best person to make them?

*III. Thought processes when making own decisions*

- Now I would like you to think back to when you made decisions for yourself. Could you walk me through that process?
- How did this compare to when you made decisions for your partner? Do you think you made different decisions for you than for your partner?
- Would you want your partner to be making these decisions for you?
- In real life, do you think you would make the same decisions that you made today?
- That is all the questions I had for you today. Is there anything that you would like to add?
