# Supplementary material for: A mixed methods investigation of end-of-life surrogate decisions among older adults
Source: BMC Palliat Care. 2020 Apr 2;19:44. doi: 10.1186/s12904-020-00553-w (PMC7119279; doi:10.1186/s12904-020-00553-w)
Supplement: Supplementary file 3 — Additional file 3: Supplementary File 3. Quantitative analysis with full sample. [file 12904_2020_553_MOESM3_ESM.docx]

Quantitative analysis with full sample

*Self-other differences****.*** Participants’ indifference points were entered into a 2 (recipient) x 3 (outcome) repeated-measures ANOVA (Figure S1). The main effect of recipient was close to significance (*F_1,23_*=3.586, *MS_e_=*697.101, *p*=.071, *η_p_^2^=*0.135). We found a main effect of outcome (*F_2,46_*=24.843, *MS_e_=*290.187, *p*<.001, *η_p_^2^=*0.519). A simple effects analysis showed that participants were more likely to accept treatment in the death scenario than in the functional impairment scenario (mean difference=-15.625, *p*<.001) and the cognitive impairment scenario (mean difference=-24.167, *p*<.001). Participants were also more likely to accept treatment in the functional impairment scenario than in the cognitive impairment scenario (mean difference=-8.542, *p*<.001). The interaction between recipient and outcome was close to significance (*F_2,46_*=3.119, *MS_e_=*114.221, *p*=.054, *η_p_^2^=*0.119) and followed a significant linear trend (*F_1,30_*=4.452, *MS_e_=*134.783, *p*=.046, *η_p_^2^=*0.162).


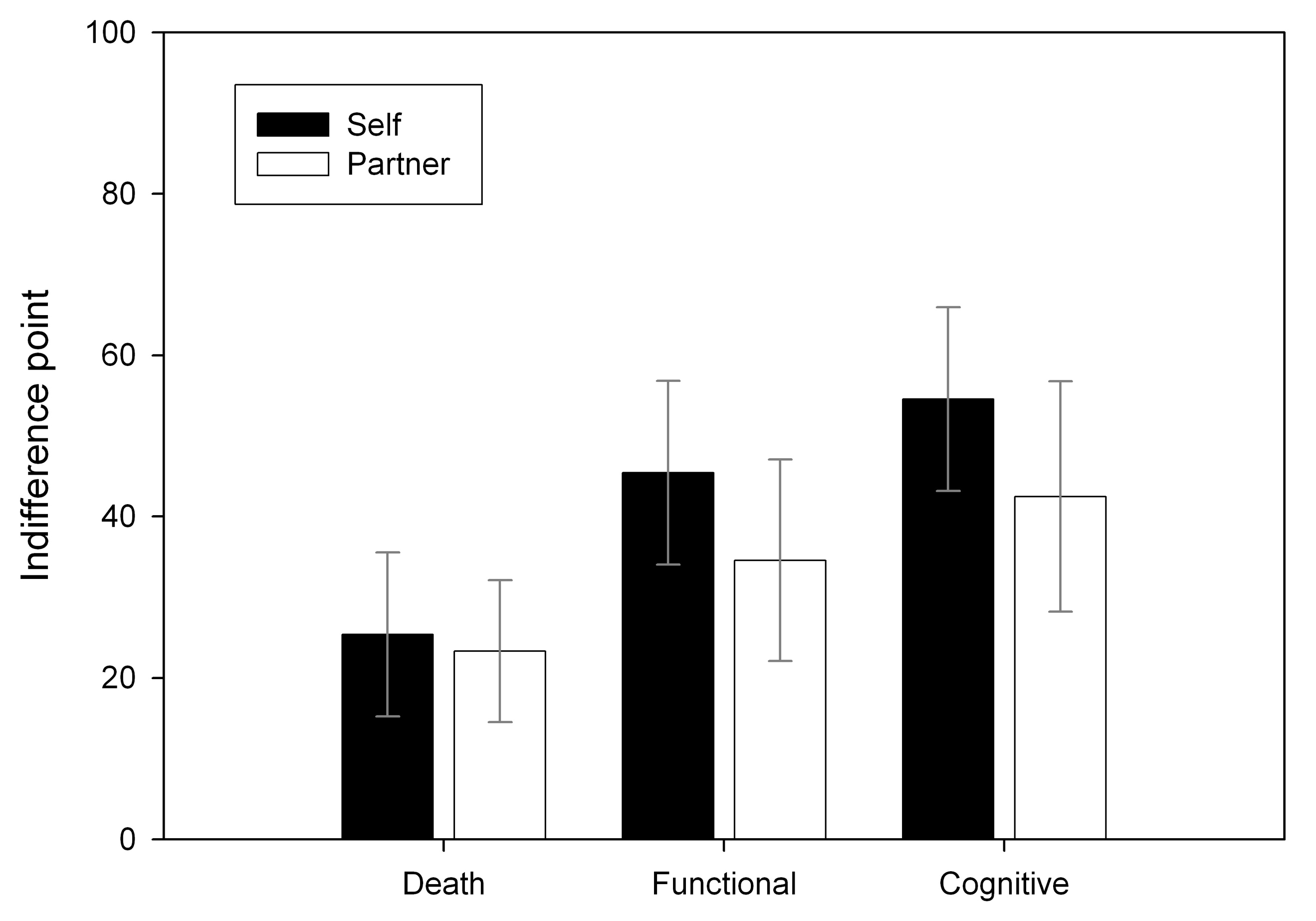


Figure S1: Participants’ indifference points for themselves versus their partner for each treatment outcome. Lower values indicate that participants were willing to accept a treatment with a lower chance of recovery and a higher risk of reduced quality of life. Error bars represent 95% confidence intervals.

*Surrogate accuracy.* We examined whether these values significantly deviated from 0 using one-sample t-tests (see Figure S2). This was the case for death (*t_23_*=3.904, *p*=.001), functional impairment (*t_23_*=7.000, *p*<.001) and cognitive impairment (*t_23_*=6.544, *p*<.001) scenarios. To investigate whether accuracy differed by scenario, we conducted a repeated-measures ANOVA with outcome as a three-level factor. We found a main effect of outcome (*F_2,46_*=4.531, *MS_e_=*314.795, *p*=.016, *η_p_^2^=*0.165). Pairwise comparisons showed that accuracy in the death scenario was higher than in the functional impairment scenario (mean difference=-12.917, *p*=.017), as well as higher than in the cognitive impairment scenario (mean difference=-13.750, *p*=.026). Accuracy between the functional and cognitive impairment scenarios did not differ (*p*=.853). Surrogate decisions were less likely to be accurate when the outcome involved living with a reduced quality of life rather than death.


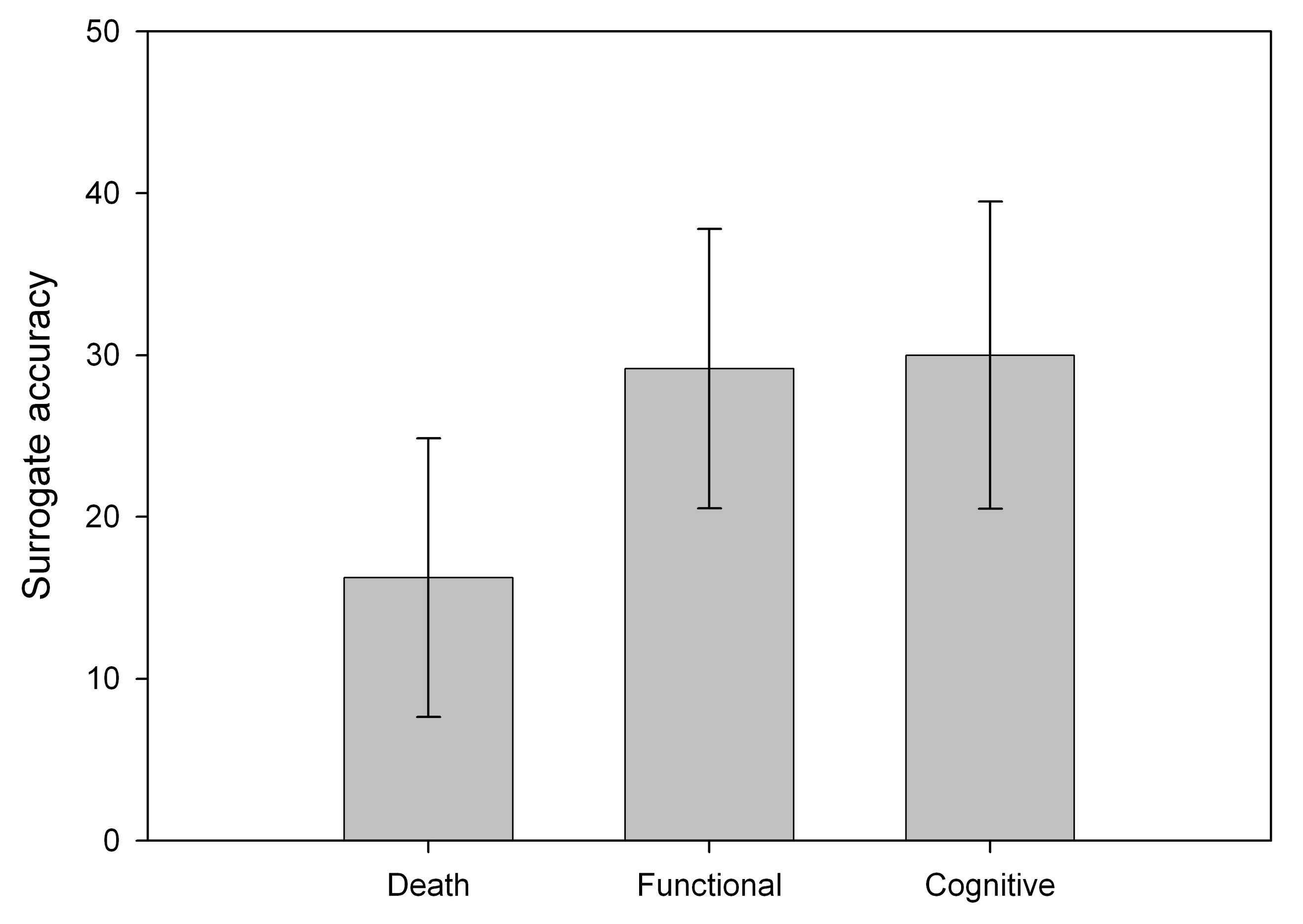


Figure S2: The deviation between surrogate decisions and the recipient’s decisions. Error bars represent 95% confidence intervals. Higher values indicate that surrogate decisions were less accurate.
